# Adsorption of lanthanide double-decker phthalocyanines on single-walled carbon nanotubes: structural changes and electronic properties as studied by density functional theory

**DOI:** 10.1007/s00894-023-05557-w

**Published:** 2023-04-26

**Authors:** Lina M. Bolívar-Pineda, Carlos Uriel Mendoza-Domínguez, Vladimir A. Basiuk

**Affiliations:** 1 Instituto de Ciencias Nucleares, Universidad Nacional Autónoma de México, Circuito Exterior C.U, Ciudad de México, 04510 México; 2 Zernike Institute for Advanced Materials, University of Groningen, Nijenborgh 4, Groningen, 9747 AG The Netherlands

**Keywords:** Lanthanides, Double-decker phthalocyanines, Carbon nanotubes, Adsorption, Density functional theory

## Abstract

**Context:**

Molecular modeling of carbon nanotubes and lanthanide double-decker phthalocyanines hybrids is challenging due to the presence of 4*f*-electrons. In this paper, we analyzed the trends in structural changes and electronic properties when a lanthanide (La, Gd, and Lu) bisphthalocyanine molecule is adsorbed on the surface of two single-walled carbon nanotubes (SWCNTs) models: armchair and zigzag. The density functional theory (DFT) computations showed that the height of bisphthalocyanines complexes (LnPc_2_) when adsorbed on a nanotube (LnPc_2_+SWCNT) is the structural feature which is most affected by the nanotube model. The formation energy of the LnPc_2_+SWCNT hybrid depends on the metal atom and the nanotube chirality. LaPc_2_ and LuPc_2_ bind stronger to the zigzag nanotube, while for GdPc_2_, bonding to the armchair nanotube is the stronger one. The HOMO-LUMO gap energy (Egap) shows a correlation between the nature of lanthanide and the nanotube chirality. In the case of adsorption on armchair nanotube, *E*_gap_ tends to match the gap of isolated LnPc_2_, whereas for adsorption on the zigzag nanotube, it is closer to the value for the isolated nanotube model. The spin density is localized on the phthalocyanines ligands (plus on Gd in the case of GdPc_2_), when the bisphthalocyanine is adsorbed on the surface of the armchair nanotube. For bonding to zigzag nanotube (ZNT), it extends over both components, except for LaPc_2_+ZNT, where spin density is found on the nanotube only.

**Method:**

All DFT calculations were carried out using the DMol^3^ module of Material Studio 8.0 software package from Accelrys Inc. The computational technique chosen was the general gradient approximation functional PBE in combination with a long-range dispersion correction developed by Grimme (PBE-D2), the double numerical basis set DN, and the DFT semi-core pseudopotentials.

**Supplementary Information:**

The online version contains supplementary material available at 10.1007/s00894-023-05557-w.

## Introduction

The lanthanide double-decker phthalocyanine complexes (also known as bisphthalocyanines) are composed of (usually) a trivalent lanthanide atom (Ln^3+^), which is coordinated with a dianionic macrocycle Pc^2-^ and a monoanionic radical ligand Pc^•-^ (that is, [Ln^3+^(Pc^2-^)(Pc^•-^)]; hereafter LnPc_2_) [1–5]. This class of complexes has attracted great interest due to their remarkable electronic and optical properties, and especially because of their single-molecule magnet (SMM) behavior. In fact, they show a large magnetic anisotropy, slow relaxation of the magnetic moment, and quantum tunneling of magnetization, which makes them promising candidates for applications in spintronics and quantum computing. These molecular quantum magnets offer the spin degree of freedom that can be used to control the charge transport in conducting systems [6, 7].

The self-assembly of LnPc_2_ on different surfaces is of particular interest, since their deposition compared to transition metal SMMs suggests the survival of a large spin magnetic moment of the rare-earth metal center [8]. LnPc_2_ complexes have been deposited onto the surfaces of copper (111) [8], gold (111) [9–12], nickel [13, 14], glass [15], and carbon nanomaterials such as graphene [7, 16], highly oriented pyrolytic graphite (HOPG) [17], and carbon nanotubes (CNTs) [4, 6, 18–21]. Unfortunately, the properties of SMM films usually change depending on the noble metal and ferromagnetic substrates or the fabrication conditions. The substrate temperature and deposition rate affect the thermodynamics and kinetics for the growth of organic films [15].

In view of their inclusion in spintronic devices, hybrids of LnPc_2_ with carbon nanomaterials such as graphene and CNTs received special attention, because there the weak spin-orbit coupling is expected to result in long spin coherence lifetimes and lengths [7]. In the case of CNTs, the noncovalent interaction with lanthanide double-decker phthalocyanines (LnPc_2_+CNTs) thorough π-π stacking is a way to improve the magnetic measurements and bistability of SMMs because the main magnetic properties of rare-earth metal center are preserved [18]. Despite this, LnPc_2_+CNTs hybrids either obtained by covalent or non-covalent functionalization of the carbon nanotubes surface have been the least explored both experimentally and computationally, contrary to transition metal phthalocyanines (monophthalocyanines, also called single-decker phthalocyanine, MPc; M(II) = Mn, Fe, Co, Ni, Cu, Zn) [4, 6, 18–21].

In our earlier work [22] dealing with rare-earth double-decker phthalocyanines, we studied the non-covalent interaction of unsubstituted yttrium bisphthalocyanines (YPc_2_) with single-walled carbon nanotubes (SWCNTs) by density functional theory (DFT; namely, by using the Perdew-Burke-Ernzerhof functional, PBE, and Grimme’s dispersion correction), analyzed structural changes in the two Pc ligands of YPc_2_ resulting from π-π interaction with the nanotube sidewalls. Other aspects we addressed were the changes in electronic characteristics upon the YPc_2_ adsorption, the effect of nanotube chirality and of the size of double numerical basis sets available in DMol^3^ module (DN, DND, and DNP). Compared to MPc and YPc_2_ hybrids with nanotubes [22–24], graphene [25, 26], and fullerenes [27, 28], the same computational task for systems including lanthanide derivatives is computationally much more demanding. As in the case of YPc_2_, a second large-size C_32_H_16_N_8_ ligand is present, which, when combined with the computational challenge of the 4f electrons from Ce to Lu, and thus with the appearance of a series of highly degenerate states, dramatically complicates the self-consistence field (SCF) convergence.

In order to proceed with DFT studies of non-covalent hybrids of LnPc_2_ complexes with carbon nanomaterials, it is crucial to obtain the optimized bisphthalocyanine structures, which represent as closely as possible to the ones obtained experimentally by X-ray diffraction (XDR). We focused on this aspect in our previous report [29]. As opposed to what could be logically expected, the larger DND and DNP basis sets (having polarization functions) were found not to be the best choice for the above purpose, due to (often) unresolvable SCF problems and distorted LnPc_2_ geometries (for example, an eclipsed conformation instead of the typical staggered one). Only the use of a smaller DN basis set helped to complete computations for all lanthanides from La to Lu, as well as to obtain reasonable LnPc_2_ geometries. Another recent study [30] on other lanthanide-containing systems (endohedral Ln@C_60_ fullerenes) showed that the use of DN and DND bases yields essentially the same geometrical and electronic features, as with the ytrium bisphthalocyanine system mentioned above. Therefore, to achieve the main goal of the present work, consisting in the analysis of structural changes and electronic properties of LnPc_2_ phthalocyanines (represented by LaPc_2_, GdPc_2_ and LuPc_2_) in LnPc_2_+SWCNTs non-covalent hybrids, we employed the DN basis set along with the PBE-D2 functional.

## Computational methods

The geometry optimizations and calculations of energies and electronic characteristics of LnPc_2_+SWCNTs hybrids were performed by using the numerical-based DFT module DMol^3^ available as part of the Materials Studio 8.0 software from Accelrys, Inc. [31–34]. The general gradient approximation (GGA) functional by Perdew-Burke-Ernzerhof (PBE) [35] in combination with a long-range dispersion correction by Grimme [36] (PBE-D2) was the computational technique of choice, because dispersion interactions need to be taken into account, when noncovalently bonded molecular systems are analyzed such as the complexes of tetraazamacrocyclic (including porphyrins and Pcs) and many other compounds with fullerene [27, 37], graphene [25, 26] and carbon nanotube models [22–24, 38, 39]. Moreover, there are already theoretical studies involving specifically MPc_2_ complexes that employed this functional [9, 40–43]. As in a recent study by our group on the optimization of geometry of lanthanide bisphthalocyanines [29], in all calculations, we employed the DFT semi-core pseudopotentials (DSPP; specially designed to use within DMol^3^ module), which implement relativistic effects and spin-orbit coupling, and the double numerical basis set DN, without polarization functions included (equivalent of 6-31G). A global orbital cutoff was set to 4.3 Å (defined by the presence of Ln atoms), and the convergence criteria were as follows: energy gradient, 10^-5^ Ha; maximum force, 0.02 Ha/Å; maximum displacement 0.05 Å; SCF tolerance, 10^-4^; and maximum step size 0.1 Å. As an auxiliary tool to facilitate SCF convergence [29], thermal smearing was used with a target value of 10^-4^ Ha (equivalent temperature of 31.6 K).

The formation energies Δ*E*_LnPc2+SWCNT_ (hereafter Δ*E* for simplicity) for the noncovalent hybrids of LnPc_2_ with SWCNTs models were calculated according to the general equation:

Δ*E*_LnPc2+SWCNT_ = *E*_LnPc2+SWCNT_ – (*E*_LnPc2_ + *E*_SWCNT_)

where *E*_*i*_ is the corresponding absolute energy.

## Results and discussion

### Structural characteristics

**Fig. 1:**
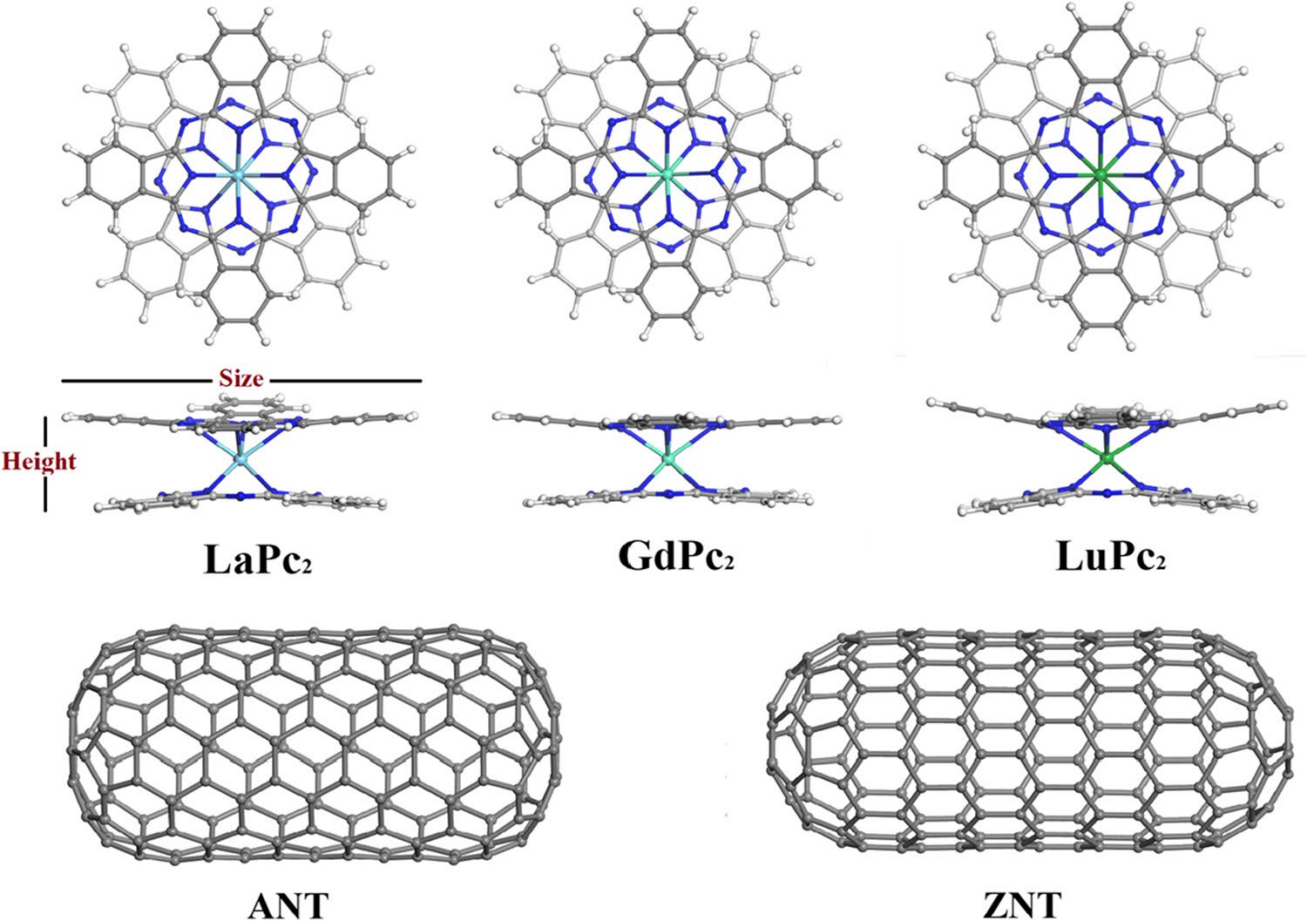
Optimized geometries for lanthanide double-decker phthalocyanines LnPc_2_ (Ln= La, Gd and Lu) and carbon nanotubes models with armchair (ANT) and zigzag (ZNT) chirality. Atom colors: gray, carbon; white, hydrogen; deep blue, nitrogen; light blue, lanthanum; turquoise blue, gadolinium; green, lutetium

To analyze the structural characteristics and electronic properties of LnPc_2_+SWCNTs hybrids, the geometry of each isolated component was optimized first. Two single-walled carbon nanotube models of different chirality were employed: armchair and zigzag, referred to as ANT and ZNT, which are composed of 180 carbon atoms with 8.23 and 7.67 Å diameter and 17.05 and 18.60 Å length, respectively, and whose ends are capped with fullerene hemispheres (Fig. 1). As representative LnPc_2_ complexes, we considered the species with a totally empty (LaPc_2_, electronic configuration [Xe]4*f* ^0^), a half-filled (GdPc_2_, [Xe]4*f* ^7^) and a totally filled (LuPc_2_, [Xe]4*f* ^14^) 4*f* shell. It was impossible to complete the geometry optimization of LnPc_2_+SWCNTs hybrids without the use of thermal smearing, but the value of 10^-4^ Ha applied here is very low (equivalent temperature of 31.6 K). At the same time, the structure optimization for LaPc_2_, GdPc_2_ and LuPc_2_ was afforded also with Fermi occupancy [29].

Structural comparison of isolated phthalocyanines and those adsorbed on the surface of nanotubes [22–24] and other carbon nanomaterials such as the endohedral fullerene Sc_3_N@C_80_ [37] and graphene with defects [25, 26] revealed an important typical feature of these macrocycles, namely, a strong bending distortion of Pc ligands upon interaction, increasing in such a way the area of Pc contact with the latter: in particular, this was observed for free-base H_2_Pc, its *3d* transition metal(II) complexes, as well as yttrium double-decker phthalocyanine interacting with SWCNTs models. This bending due to non-parallel π-π interactions between the two extended π systems occurs to a variable degree, depending on the central atom, the diameter and chirality of the carbon nanotubes [22–24, 44, 45]. The structural parameters we used to characterize such a distortion for each LnPc_2_ in the isolated and the adsorbed state in LnPc_2_+SWCNTs hybrids (Table 1) are the rotation angle between the two Pc ligands (skew angle; φ), the molecular size (width), height, the N-Ln distance, and the N-Y-N angles. In the case of isolated LnPc_2_ species, each parameter was compared with the one found in experimentally derived structure XRD from [46, 47] (details of such comparisons were described in Ref. [29]). Also, the formation energies, HOMO, LUMO and HOMO-LUMO gap energies (*E*_gap_), the charge and spin of central metal atom (Table 2), as well as the spin density distribution, were analyzed.

**Table 1:** Size (in Å), height (in Å), Ln-N bond length (Å), and N-Ln-N angle (in degrees) for isolated LnPc_2_ molecules and for LnPc_2_ bound noncovalently to a single-walled carbon nanotube (LnPc_2_+SWCNTs with SWCNTs = ANT and ZNT), as well as the shortest distances: Ln^…^C_SWCNT_, γ-N^…^C_SWCNT_ and C_LnPc2_^…^C_SWCNT_ (in Å) between LnPc_2_ and SWCNT, calculated using the PBE GGA functional with Grimme’s dispersion correction in conjunction with DN basis set. The structural parameters for the crystal structure of LnPc_2_ obtained from X-ray diffraction [46, 47] are listed for comparison

System	Height (Å)	Size (Å)	N-Ln-N (°)	Ln-N (Å)	Ln^…^C_SWCNT_(Å)	γ-N^…^C_SWCNT_(Å)	C_LnPc2_^…^C_SWCNT_(Å)
LaPc_2_^a^	3.936-4.381	14.703	108.8-108.9	2.421			
GdPc_2_^a^	3.652-4.912	14.679-14.733	108.2-108.8	2.422-2.436			
LuPc_2_^a^	3.743-5.182	14.599-14.717	110.8-112.0	2.358-2.388			
LaPc_2_	3.803-5.414	15.126-15.170	107.6-107.3	2.536-2.545			
GdPc_2_	3.858-4.558	15.098-15.132	107.1-107.6	2.507-2.529			
LuPc_2_	4.358-5.010	14.925-15.046	114.1-114.2	2.384-2.400			
LaPc_2_+ANT	4.087-5.805	14.838-15.151	107.3-107.8	2.522-2.553	4.551	3.107	3.160
GdPc_2_+ANT	3.920-4.566	15.043-15.147	111.1-113.2	2.405-2.444	4.597	3.180	3.279
LuPc_2_+ANT	4.591-5.193	14.835-15.052	113.6-114.4	2.379-2.405	4.619	3.231	3.218
LaPc_2_+ZNT	3.899-5.998	14.863-15.091	107.3-108.7	2.519-2.550	4.692	3.180	3.129
GdPc_2_+ZNT	4.320-4.911	14.839-15.143	112.4-114.0	2.411-2.430	4.559	3.192	3.089
LuPc_2_+ZNT	4.189-5.289	14.876-15.030	113.6-115.7	2.364-2.391	4.533	3.123	3.076

^a^ XRD data

**Table 2:** Total energies *E*_*total*_ (in Ha), formation energies ΔE (in kcal/mol), HOMO, LUMO and HOMO-LUMO gap energies (in eV) for the isolated LnPc_2_, SWCNTs (ANT and ZNT) and for the noncovalently hybrids, as well as charge and spin of Ln (La, Gd and Lu) and charge transfer from LnPc_2_ to SWCNTs calculated using PBE-GGA functional with Grimme’s dispersion correction in conjunction with the DN basis set. The charge transfer values were obtained from Mulliken population analysis

System	*E*_total_ (Ha)	Δ*E* (kcal/mol)	HOMO (eV)	LUMO (eV)	*E*_gap_ (eV)	Ln charge (*e*)	Charge on LnPc_2_ (*e*)^a^	Ln spin (*e*)
ANT	-6852.4098123		-5.417	-4.866	0.551			
ZNT	-6852.3450966		-5.735	-5.734	0.001			
LaPc_2_	-3388.1293982		-4.845	-4.713	0.133	1.893		0
GdPc_2_	-3527.7074389		-4.811	-4.680	0.130	1.532		7.006
LuPc_2_	-3981.3338903		-4.683	-4.545	0.138	1.438		0.002
LaPc_2_+ANT	-10240.6227541	-52.4	-5.063	-4.935	0.128	1.924	0.090	0
GdPc_2_+ANT	-10380.2217485	-65.6	-4.861	-4.730	0.131	1.487	0.082	7.010
LuPc_2_+ANT	-10833.8314531	-55.1	-4.873	-4.740	0.134	1.457	0.079	0.002
LaPc_2_+ZNT	-10240.5628015	-55.4	-5.345	-5.324	0.021	1.992	0.377	0
GdPc_2_+ZNT	-10380.1555445	-64.6	-5.232	-5.217	0.014	1.472	0.452	7.013
LuPc_2_+ZNT	-10833.7757678	-60.7	-5.260	-5.248	0.012	1.431	0.502	0

^a^ The charge transfer is always from LnPc_2_ to SWCNT model

As most unsubstituted double-decker phthalocyanines, the ones studied in this work are characterized by a staggered structure (Fig. 1), where the mutual rotation angles between Pc ligands approaches 45° [3, 29]. The corresponding values for the optimized LaPc_2_ and GdPc_2_ complexes do not show tangible differences compared to the experimental XRD structures (0.38° and 0.09°, respectively), whereas for LuPc_2_ this angle differs by 4.9°. This discrepancy can be attributed to the fact that the experimental value refers to the crystalline phase while the theoretical approach considers an isolated molecule, as well as the presence of solvent in the crystal lattice (the reported LuPc_2_ structure included [NBu_4_]^+^ cation, creating very particular chemical environment [47]). In the phthalocyanines adsorbed on the surface of SWCNTs models, the skew angles are not affected: their values vary between 44.8° and 44.9° only, depending on LnPc_2_ complex and nanotube chirality.

The size (or width) of LnPc_2_ molecules, which is defined as the maximum distance between the two hydrogen atoms at opposite *o*-phenylene moieties of Pc rings [11, 48], is overestimated in all cases (Table 1), fluctuating around 15.0 Å. Hence, the length of the nanotube models is barely sufficient to accommodate one LnPc_2_ molecule. As seen in Table 1, the distance between the hydrogen atoms is not the same for all LnPc_2_ complexes. Upon adsorption on the SWCNTs models, the *o*-phenylene moieties are attracted to the nanotube sidewall, leading to a more domed geometry of Pc ligand contacting SWCNTs. The bending distortion is more noticeable when bisphthalocyanines are adsorbed on ZNT, since its diameter is smaller than that of ANT.

It is known that the two Pc ligands of neat LnPc_2_ complexes are not planar, exhibiting a different degree of bending in the isoindole units (Fig. 1 and Ref. [29]), and this distortion is attributed to the repulsive interaction between the two macrocycles, especially between the *o*-phenylene rings. A quantitative evaluation of this distortion can be made by analyzing the height of each LnPc_2_ complex, which is measured as the distance between the peripheral hydrogen atoms belonging to the opposing Pc ligands [11, 49]. The height of La, Gd, and Lu bisphthalocyanine as well as their size (or width) vary within the same molecule, so that it is usually presented as an interval within which the above H^...^H distances are found (Table 1). For XRD structures of LaPc_2_, GdPc_2_, and LuPc_2_, these intervals are respectively 3.936–4.381 (variation within 0.445 Å), 3.652–4.912 (variation within 1.260 Å), and 3.743–5.141 Å (variation within 1.439 Å). For DFT-optimized LnPc_2_ geometries, the height variation increases for LaPc_2_ (from 0.445 to 1.611 Å), but decreases for GdPc_2_ (from 1.260 to 0.700 Å) and LuPc_2_ (from 1.439 to 0.652 Å). When LnPc_2_ complexes interact with SWCNTs models, the molecule height increases, especially in LnPc_2_+ZNT hybrids (except for GdPc_2_+ZNT, the H^...^H distances in decreases). The most noticeable change compared to the height calculated for isolated molecules is observed for La and Lu bisphthalocyanines, for which the height variation increases by about 0.45 Å. This suggests that the height is affected by bending distortion of Pc ligand contacting nanotube sidewall, as a result of strong π-π interactions between the two components.

**Fig. 2:**
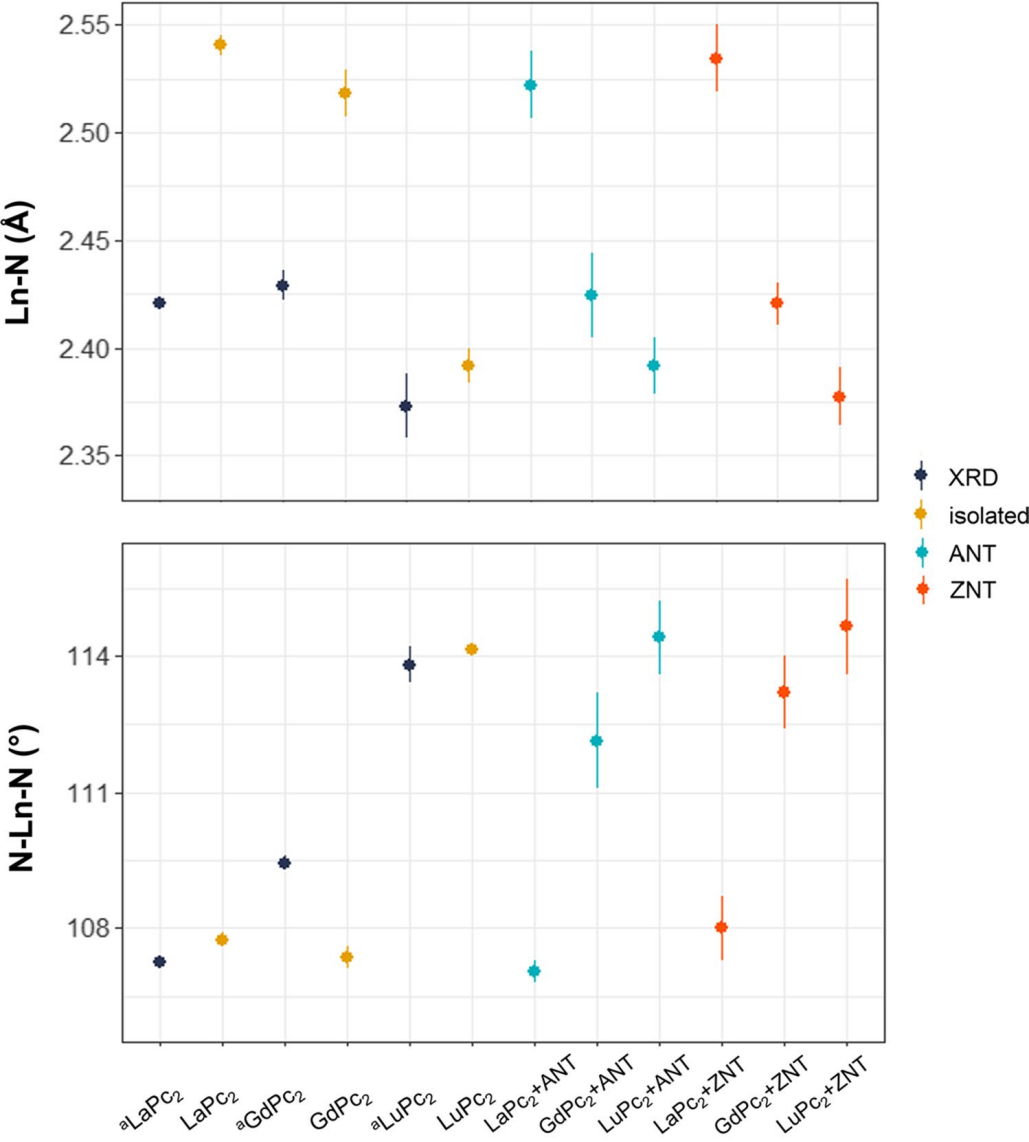
Comparison of the Ln-N bond lengths (Å; top) and N-Ln-N angles (°; bottom) in crystalline lanthanide double-decker phthalocyanine complexes obtained by XRD (^a^LnPc_2_), in the isolated LnPc_2_ molecules and adsorbed on the carbon nanotube sidewalls (LnPc_2_+SWCNTs) calculated at the PBE-D2/DN level of theory

Another set of parameters, which can be employed to evaluate the distortion of LnPc_2_, is the length of coordination bonds between the lanthanide and nitrogen atoms of isoindole units (Ln-N). The calculated Ln-N bond lengths in isolated LnPc_2_ molecules are 2.536–2.545 Å for LaPc_2_, 2.507–2.529 Å for GdPc_2_, and 2.384–2.400 Å for LuPc_2_, and hence larger than the XRD experimental values (Table 1 and Fig. 2). Figure 2 shows how the Ln-N length in each isolated bisphthalocyanine decreases as the Ln atomic number increases, and that this trend is maintained after adsorption on the nanotube sidewall. The N-Ln-N angles in isolated complexes were analyzed as well (Table 1 and Fig. 2), and result underestimated for GdPc_2_, and overestimated for LaPc_2_ and LuPc_2_, compared to the experimental values. The angles of most of the bisphthalocyanines increase after deposition on the surface of each model nanotube with respect to the isolated and optimised structure and reflect greater variation in the range, indicating asymmetry and distortion, the exception of the lanthanum double-decker phthalocyanine on the surface of armchair nanotube, the value of the angles decreases, see Table 1 and Fig. *2*. For LnPc_2_+SWCNTs, the change in N-Ln-N angle is opposite to that of Ln-N bond lengths: the angles increase from La to Lu. The change become more dramatic in the gadolinium hybrids.

The attraction between the SWCNTs models and LnPc_2_ complexes can be characterized in terms of the shortest Ln^…^C_SWCNT_, γ-N^…^C_SWCNT_ and C_LnPc2_^…^C_SWCNT_ distances. For LaPc_2_+ANT and GdPc_2_+ANT closest distance is found between a carbon atom of the nanotube and one of the azomethine nitrogen atoms (γ-N) of the Pc ligand (γ-N^...^C_SWCNT_; 3.107 and 3.180 Å_,_ respectively), meanwhile for LuPc_2_+ANT and all three LnPc_2_+ZNT hybrids, the closest contact is between carbon atoms, C_LnPc2_^…^C_SWCNT_. The shortest distance between lanthanide and a carbon atom of the nanotube (Ln^...^C_SWCNT_; Table 1) is one of the structural parameters that is most sensible to the nanotube model and the Ln species. In the LnPc_2_+ANT series, this distance increases as the lanthanide atomic number increases, from 4.551 Å for LaPc_2_+ANT to 4.619 Å for LuPc_2_+ANT, while an opposite behavior is observed for the LnPc_2_+ZNT series, where it decreases from 4.692 Å for LaPc_2_+ZNT to 4.533 Å for LuPc_2_+ZNT.

It is important to mention that the use of electron smearing technique, as a tool to solve the SCF convergence problems [29, 50–52], does not affect the geometry features for isolated LnPc_2_ complexes, compared to those computed using Fermi occupancy, when the smearing values are as low as (1-5)x10^-4^ Ha.

### Adsorption strength and electronic properties

From Table 2, one can see that the complex formation energy (or adsorption energy) depends on the nature of metal. The lowest negative Δ*E* values of −65.6 and −64.6 kcal/mol were obtained for GdPc_2_+ANT and GdPc_2_+ZNT, respectively, indicative of the strongest binding. For both the ANT and the ZNT series, Δ*E* increases in the order of GdPc_2_ < LuPc_2_ < LaPc_2_. Concerning the effect of the nanotube chirality, LaPc_2_ and LuPc_2_ adsorbed on ZNT show more negative energies than the ones adsorbed on ANT: −55.4 and −60.7 kcal/mol vs*.* −52.4 and −55.1 kcal/mol, respectively. At the same, an opposite trend can be seen for GdPc_2_+SWCNTs hybrids, though the difference is as small as 1 kcal/mol. In this regard, it is interesting to mention that LaPc_2_+SWCNTs behave similarly to their YPc_2_ analogues [22], where the central rare-earth metal has no *f*-orbitals, and the nanotube models were substantially smaller.

**Fig. 3:**
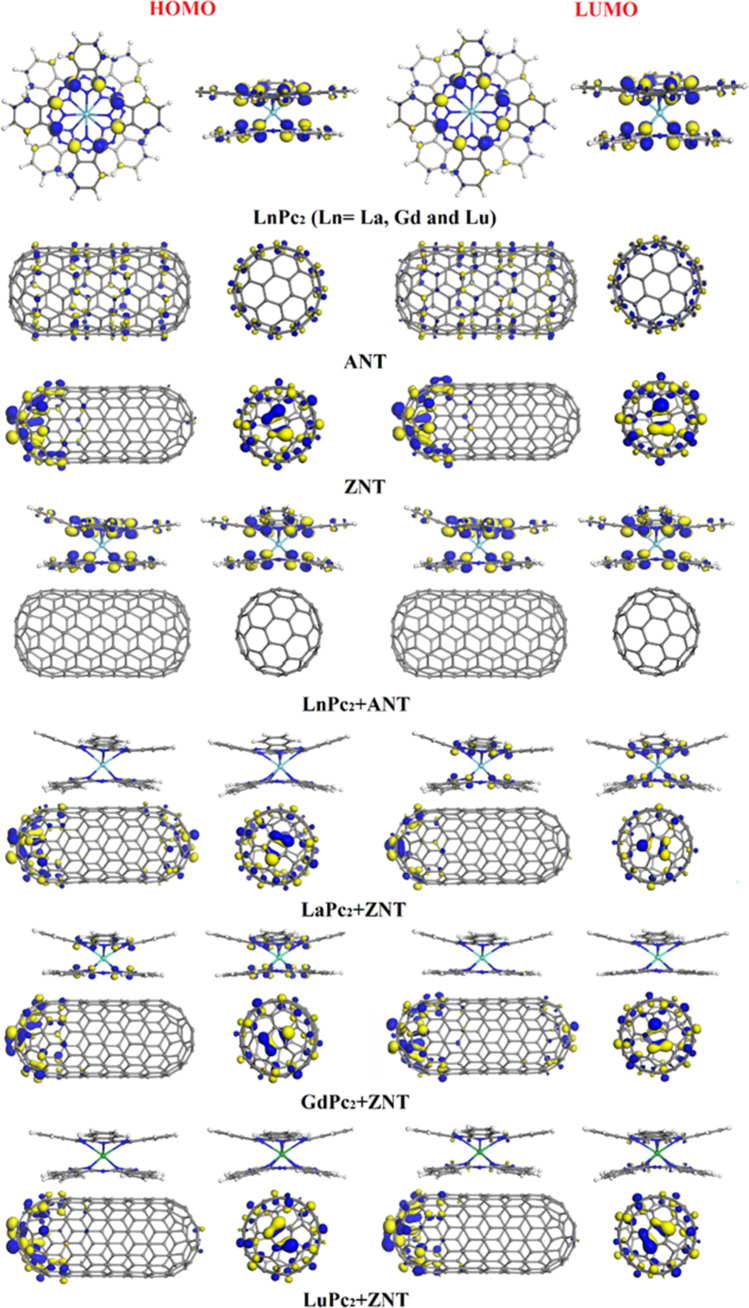
HOMO and LUMO plots (isosurfaces at 0.03 a.u; two side views) for lanthanide double-decker phthalocyanines (LaPc_2_, GdPc_2_ and LuPc_2_), SWCNTs models, and LnPc_2_+SWCNTs hybrids calculated by using the PBE GGA functional with Grimme’s dispersion correction with the DN basis set

We also calculated HOMO, LUMO and HOMO-LUMO gap energies (Table 2), and analyzed the corresponding frontier orbital plots (Fig. 3). The gap energy for isolated LnPc_2_ complexes slightly decreases in the order of LuPc_2_ (0.138 eV)> LaPc_2_ (0.133 eV)> GdPc_2_ (0. 130 eV), as in earlier calculations with Fermi occupancy [22]. Among the nanotube models, ANT exhibits a higher band gap than ZNT (0.551 and 0.001 eV, respectively), similarly to the smaller nanotube models with the same chirality used to study their non-covalent interactions with 3*d* transition metal(II) MPcs [22, 23, 39] and YPc_2_ [24]. For what concerns the gap energy of the LnPc_2_+SWCNTs hybrids, the following observations can be made. Firstly, for LnPc_2_+ANT hybrids *E*_gap_ changes linearly with the lanthanide atomic number. The gap becomes slightly larger as the atomic number, and consequently the number of 4*f*-electrons increases: 0.128 eV for LaPc_2_, 0.131 eV for GdPc_2_, and 0.134 eV for, LuPc_2_. For LnPc_2_+ZNT, trend is opposite, but the *E*_gap_ values are smaller by one order of magnitude: 0.021, 0.014 and 0.012 eV for LaPc_2_, GdPc_2_ and LuPc_2_, respectively. Secondly, comparing the computed gap values of each hybrid with that of the isolated component (Table 2), one can conclude that in the case of LnPc_2_+ANT, *E*_gap_ tends to approach the one of the respective isolated LnPc_2_, whereas in the LnPc_2_+ZNT series, it is closer to the band gap of the nanotube, a feature that was found also for YPc_2_+SWCNTs dyads [24]. The fact that the gap energy is higher for LnPc_2_+ANT than for LnPc_2_+ZNT dyads, also observed in our earlier studies of hybrids with 3*d* transition metal(II) MPcs [22, 23, 39] and YPc_2_ [24], can be interpreted as an effect of the nanotube chirality. At the same time, our theoretical band gap values should be taken with a certain precaution, since it is known that they are strongly underestimated when using pure GGA functionals (PBE in particular).

As far as the distribution of frontier orbitals is concerned, Fig. 3 and Fig. S1 illustrate that for isolated LaPc_2_, GdPc_2_, and LuPc_2_, the HOMO and LUMO is localized on the carbon atoms of the macrocycle, specifically at the pyrrole unit, as observed earlier for YPc_2_ [24] and LnPc_2_ [29] by us and by other research groups at different theoretical levels [40, 53]. For LnPc_2_+SWCNTs hybrids, its behavior depends on the nanotube chirality and on the central Ln atom (Fig. 3 and Fig. S2). In the hybrids with ANT, HOMO and LUMO are localized on bisphthalocyanine as in isolated LnPc_2_ and in YPc_2_+ANT [22]. In LnPc_2_+ZNT, the frontier orbital distribution varies. In LaPc_2_+ZNT and LuPc_2_+ZNT, HOMO is located exclusively on nanotube, and LUMO on both components; in the case of LuPc_2_+ZNT, the contribution from the nanotube is more notable. In GdPc_2_+ZNT, HOMO extends over both components and LUMO is localized only nanotube, similarly to the case of YPc_2_+ZNT [22]. An additional detail, which can be observed in Fig. 3, is that neither HOMO nor LUMO is localized on the central Ln metal.

One more aspect of interest we addressed is the charge of lanthanide atom (Table 2), as estimated from the Mulliken population analysis. The charge of La, Gd, and Lu in isolated bisphthalocyanines is 1.827, 1.452, and 1.400 *e*, respectively. In the case of hybridss, the changes are rather random. For LaPc_2_+SWCNTs hybrids, there is an increase by 0.031 *e* for LaPc_2_+ANT and 0.099 *e* for LaPc_2_+ZNT. For their GdPc_2_+ANT and GdPc_2_+ZNT, the Gd charge decreases by 0.045 and 0.060 *e,* respectively. For LuPc_2_+SWCNTs hybrids, the Lu charge increases by 0.019 *e* for LuPc_2_+ANT but decreases insignificantly, by 0.007 *e* for LuPc_2_+ZNT. Regardless of the magnitude, the general trend the same as for isolated phthalocyanines, where the Ln charge decreases in the order of LaPc_2_ > GdPc_2_ > LuPc_2_.

The trend of charge transfer within the hybrids was analyzed since carbon nanotubes and phthalocyanine hybrids have been considered as supramolecular self-assembled donor-acceptor conjugated systems. From Table 2, it is clear that the direction of charge transfer is from the phthalocyanine to the carbon nanotube and is influenced by the chirality of the nanotube and the central coordination metal. For phthalocyanines adsorbed on the surface of armchair nanotubes, the charge transfer increases inversely to the lanthanide atomic number, from 0.079 (LuPc_2_+ANT) to 0.090 *e* (LaPc_2_+ANT), while for zigzag nanotube hybrids, it increases directly from 0.377 (LaPc_2_+ZNT) to 0.502 *e* (LuPc_2_+ZNT), and the latter hybrids apparently generate a higher charge transfer. Something particular that can be denoted and associated, is the Ln^…^C_SWCNT_ distance, for each set of hybrids per chirality, which has opposite behavior to that structural parameter (Table 1), and that is that the smaller the Ln^…^C_SWCNT_ distance, the higher the charge transfer.

**Fig. 4:**
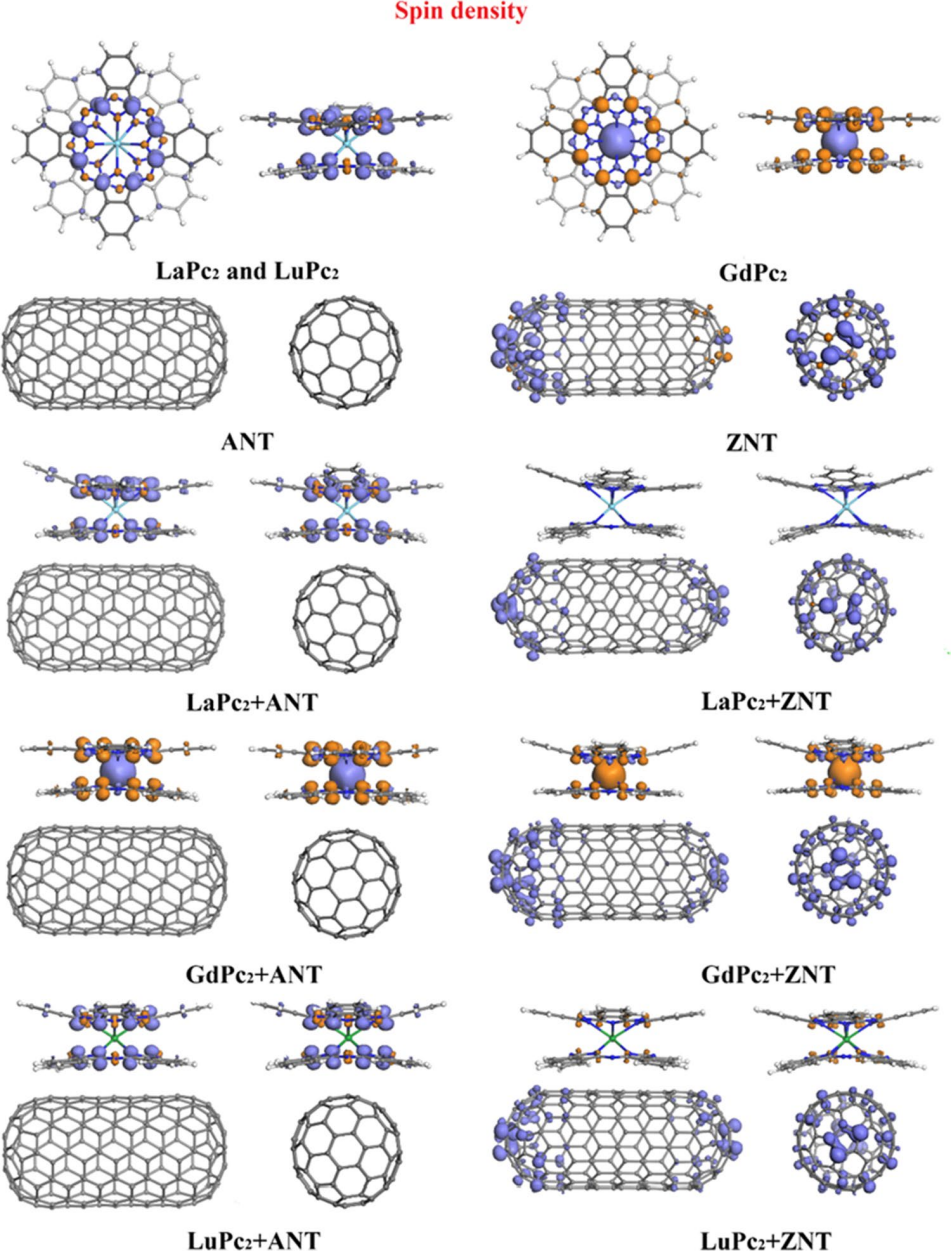
Spin density plots for lanthanides double-decker phthalocyanine (LaPc_2_, GdPc_2_ and LuPc_2_;), SWCNTs models, and LnPc_2_+SWCNTs hybrids (isosurfaces at 0.01 a.u;) calculated by using the PBE GGA functional with Grimme’s dispersion correction with the DN basis set. Violet and orange lobes correspond to spin-up and spin-down electrons, respectively

Spin density plots calculated for the isolated LnPc_2_ complexes, the SWCNTs models, and the LnPc_2_+SWCNTs hybrids are presented in Fig. 4 (also in Figs. S1 and S2). The distribution of the spin density in isolated LaPc_2_ and LuPc_2_ matches closely the HOMO and LUMO distribution discussed above (Fig. 3). In these complexes, the unpaired electrons are found mainly on carbon atoms of the pyrrole unit that are bonded with the nitrogen atoms, as well as a minor contribution from γ-N and isoindole N atoms. This feature is also present in GdPc_2_, but the additional main contribution here comes from the metal.

The spin distribution in the hybrids depends not only on the central metal, but also on the nanotube model. The plots for the LnPc_2_+ANT hybrids very similar to those of the isolated LnPc_2_ complexes, while those for the three hybrids with ZNT exhibit notable differences. In all of them, one can observe the presence of unpaired electrons on the closed nanotube ends (as in the isolated ZNT model). No tangible contribution from the bisphthalocyanine can be found in LaPc_2_+ZNT, and only a minor one in that of LuPc_2_+ZNT. This is in contrast to GdPc_2_+ZNT, where the spin density distribution of the isolated GdPc_2_ and of ZNT is combined, in the latter the contribution of spin up (violet lobule) and spin down (orange lobule) in the complex is reversed when deposited in armchair nanotubes. Although qualitatively no differences are observed in the spin density of the ligands of each phthalocyanine on the nanotubes, quantitatively it can be deduced that for LaPc_2_ on both nanotubes, the ligand that is closer to the nanomaterial wall has a lower density (for ANT 0.441 vs. 0.575 *e* and for ZNT-0.003 vs. 0.022 *e*) opposite to the behavior of GdPc_2_ (for ANT -0.445 vs. 0.569 *e* and for ZNT -0.217 vs. -0.239 *e*). Meanwhile, LuPc_2_ adsorbed on ANT the ligand has a lower density 0.419 vs. 0.533 *e*) and on ZNT a higher density (-0.113 vs. -0.143 *e*).

Table 2 specifies also the spin of Ln atoms in isolated and adsorbed double-decker phthalocyanines. One can see that the Ln spin remains relatively constant. For LaPc_2_ and LuPc_2_ complexes, where the lanthanide(III) ion is in a closed-shell configuration, it is always close to zero. On the other hand, for GdPc_2_ where the 4*f* orbital of gadolinium ion is half-filled, a minor spin transfer of 0.004 and 0.007 *e* from ANT and ZNT, respectively, was found.

## Conclusions

The main results can be summarized as follows:

- The height of LnPc_2_ complexes adsorbed on nanotubes is one of the structural features which is most affected by the SWCNTs diameter; the distance between H atoms of opposite Pc ligands increases stronger more for the nanotube with the smaller-diameter, because the ligands are more strongly bent and take on a domed geometry.
- Ln-N bond length and N-Ln-N angle of lanthanides bisphthalocyanines on nanotubes with both armchair and zigzag follow the same trend as in isolated LnPc_2_, the Ln-N bond length decreases with increasing atomic number of the central metal, while the value of N-Ln-N increases.
- The formation energy of LnPc_2_+SWCNTs hybrids depends on the type of lanthanide and the nanotube chirality. LaPc_2_ and LuPc_2_ bond stronger to the nanotube with armchair chirality than to the zigzag tube, while the opposite occurs in the case of GdPc_2_ (though the difference is insignificant).
- The HOMO-LUMO gap width correlates with the number of electrons of lanthanide and nanotube chirality. For LnPc_2_+ANT hybrids *E*_gap_ increases linearly in the order LaPc_2_ < GdPc_2_ < LuPc_2_, whereas LnPc_2_+ZNT hybrids the trend is opposite and *E*_gap_ decreases in the order LaPc_2_ > GdPc_2_ > LuPc_2_. Hybrids resulting from the adsorption on the armchair nanotube have a larger gap compared to the case when LnPc_2_ binds to zigzag tube. In the former case, *E*_gap_ tends to match the gap of isolated LnPc_2_, whereas in the latter case it is closer to the value for zigzag tube alone.
- The spin density for LnPc_2_ adsorbed on the armchair nanotube is localized on the Pc ligands, for LnPc_2_ adsorbed on the zigzag nanotube on both interaction components, and for LaPc_2_ deposited on the zigzag tube only on nanotube.

## Supplementary Information

ESM 1 (DOC 6684 kb)
