# Supplementary material for: Adsorption of lanthanide double-decker phthalocyanines on single-walled carbon nanotubes: structural changes and electronic properties as studied by density functional theory
Source: J Mol Model. 2023 Apr 26;29(5):158. doi: 10.1007/s00894-023-05557-w (PMC10133082; doi:10.1007/s00894-023-05557-w)
Supplement: Supplementary file 1 — (DOC 6684 kb) [file 894_2023_5557_MOESM1_ESM.doc]

**Supplementary information**

**Adsorption of lanthanide double-decker phthalocyanines on single-walled carbon nanotubes: structural changes and electronic properties as studied by density functional theory**

Lina M. Bolivar-Pineda,^a,b^,* [Carlos Uriel Mendoza-Domínguez](https://www.sciencedirect.com/science/article/abs/pii/S025405842100746X#!),^a^ and Vladimir A. Basiuk^a^,*

^a^ *Instituto de Ciencias Nucleares, Universidad Nacional Autónoma de México, Circuito Exterior C.U., Ciudad de México 04510, México;*

^b^ *Zernike Institute for Advanced Materials, University of Groningen, Nijenborgh 4, Groningen 9747 AG, The Netherlands*

__________________________________________________

*Corresponding authors:

E-mail: l.m.bolivar.pineda@rug.nl, [basiuk@nucleares.unam.mx](mailto:basiuk@nucleares.unam.mx)

ORCID

Lina M. Bolivar-Pineda: 0000-0003-0586-7896

**[Carlos Uriel Mendoza-Domínguez](https://www.sciencedirect.com/science/article/abs/pii/S025405842100746X#!) 0000-0003-0331-2037**

Vladimir A. Basiuk: 0000-0001-7864-9203


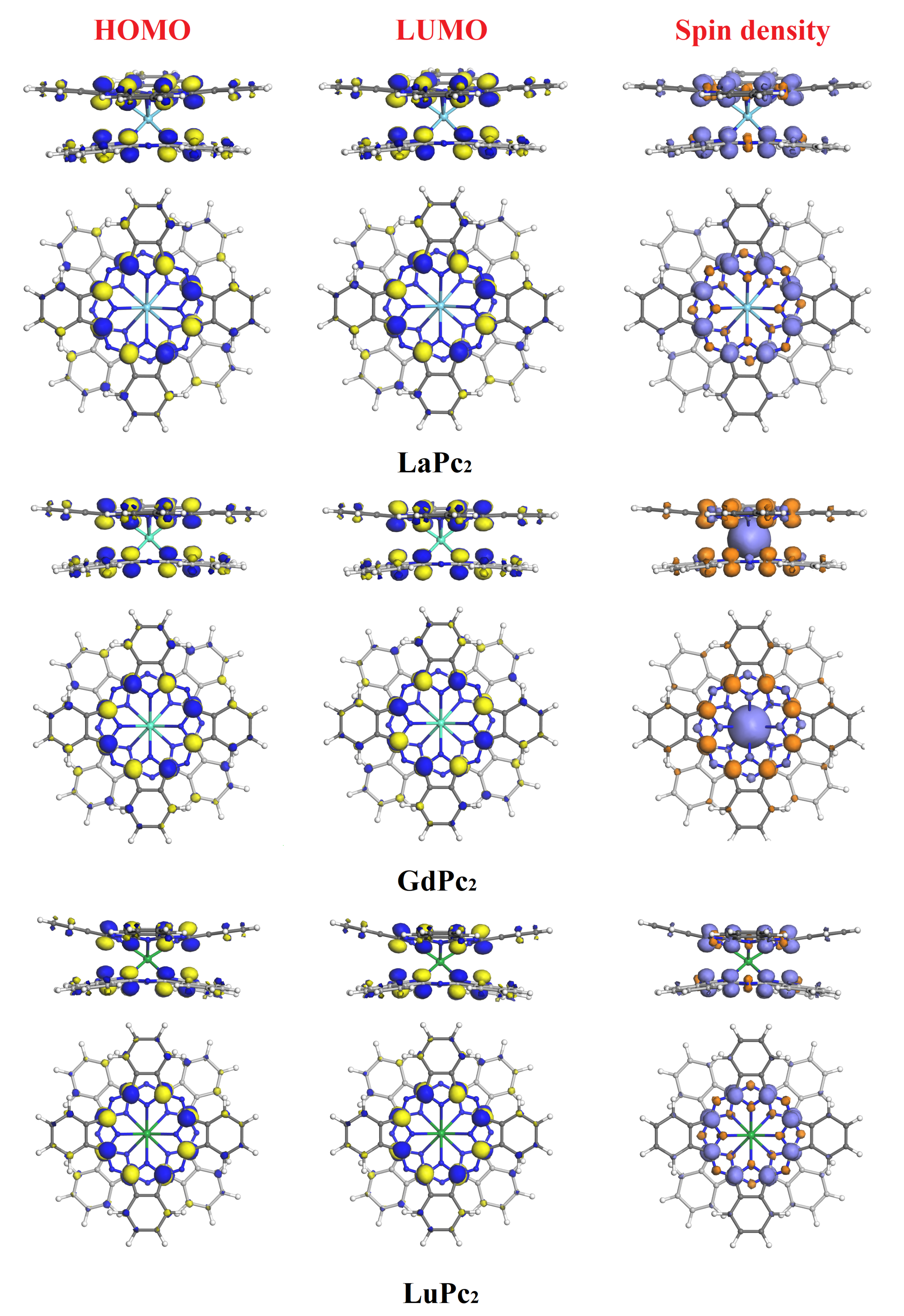


**Figure S1**. HOMO, LUMO (isosurfaces at 0.03 a.u.; side and top views) and spin density plots (isosurfaces at 0.01 a.u.) for lanthanide double-decker phthalocyanines (LaPc_2_, GdPc_2_ and LuPc_2_) calculated by using the PBE GGA functional with Grimme’s dispersion correction with the DN basis set


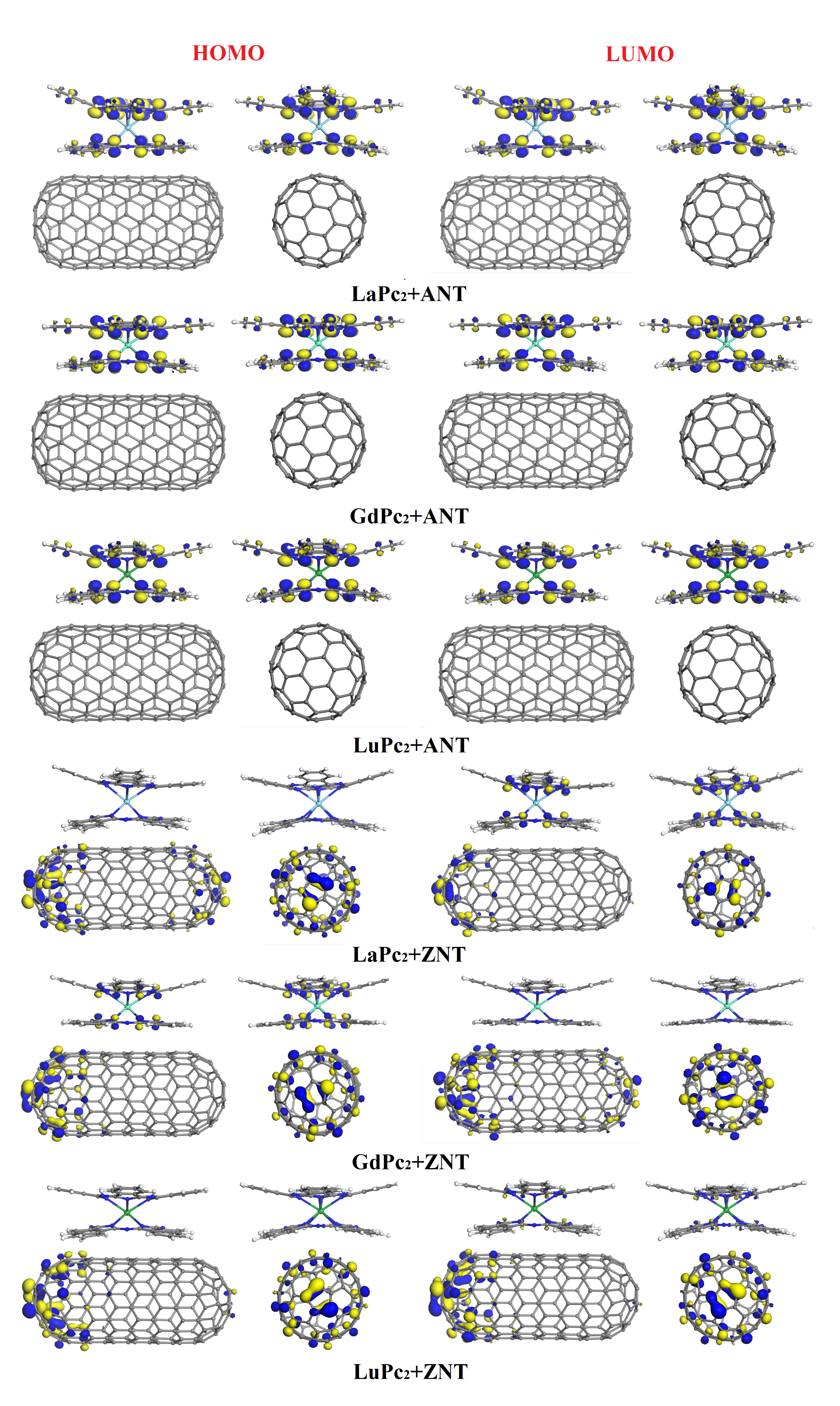


**Figure S2**. HOMO and LUMO plots (isosurfaces at 0.03 a.u; two side views) for lanthanide double-decker phthalocyanines (LaPc_2_, GdPc_2_ and LuPc_2_)on the surface of armchair and zigzag carbon nanotube models (LnPc_2_+ANT and LnPc_2_+ZNT hybrids, respectively) calculated by using the PBE GGA functional with Grimme’s dispersion correction with the DN basis set.
